# A case report on the postoperative management of phyllodes tumor of the breast and literature review

**DOI:** 10.1097/MD.0000000000043668

**Published:** 2025-08-01

**Authors:** Yuge Ran, Fei Li, Shuai Qie

**Affiliations:** a Department of Radiation Oncology, Affiliated Hospital of Hebei University, Baoding, Hebei Province, PR China; b School of Nursing, Hebei University, Baoding, Hebei Province, PR China.

**Keywords:** adjuvant radiotherapy, breast neoplasm, case report, phyllodes tumor, surgical margin

## Abstract

**Rationale:**

Phyllodes tumor of the breast (PTB) is a rare fibroepithelial neoplasm with controversial postoperative management, particularly for borderline and malignant subtypes. Limited large-scale studies exist on optimal radiotherapy strategies and recurrence prevention, necessitating further exploration of multidisciplinary approaches.

**Patient concerns:**

A 50-year-old female presented with a rapidly enlarging right breast mass (from 2 to 3 cm to 12 cm within a week), initially detected 10 years prior. Imaging revealed a BI-RADS 4B cystic-solid lesion, and biopsy confirmed a malignant neoplasm with necrosis.

**Diagnoses:**

Postmastectomy pathology confirmed a malignant PTB (T4N0M0GX) with stromal overgrowth, adipose tissue invasion, and high Ki-67 (90%+). Immunohistochemistry showed Vimentin+, CK+, and BCL2+, while ER/PR were negative.

**Interventions:**

The patient underwent right mastectomy followed by adjuvant radiotherapy targeting the tumor bed (gross tumor volume tumor bed: 60 Gy/25F) and expanded margins (planning tumor volume: 50 Gy/25F). No chemotherapy or endocrine therapy was administered due to limited evidence of efficacy.

**Outcomes:**

Postoperative recovery was uneventful, with no local recurrence observed during follow-up. Radiotherapy achieved optimal local control, aligning with studies suggesting reduced recurrence in margin-challenged cases.

**Lessons:**

This case provides critical clinical evidence supporting adjuvant radiotherapy as a viable salvage strategy for malignant PTB with close/positive margins (≤1 cm), particularly when re-excision is contraindicated. By demonstrating 12-month disease-free survival with acceptable toxicity, our findings directly address the NCCN guideline gap regarding postmastectomy radiation for borderline resectable PTB. The documented multidisciplinary decision-making process – incorporating tumor biology (high Ki-67), anatomical constraints, and patient preferences – offers a replicable framework for similar cases while underscoring the urgent need for consensus protocols through prospective registry studies.

## 1. Introduction

Phyllodes tumor of the breast (PTB) is a localized fibroepithelial tumor with a leaf-like structure, covered by luminal epithelium and myoepithelial cell layers, accompanied by an increase in stromal cells. It accounts for less than 1% of breast tumors.^[1]^ According to the latest WHO classification criteria in 2019, PTB is pathologically classified into benign, borderline, and malignant based on tumor cell atypia, nuclear mitotic figure ratio, and cell necrosis degree. The peak age of onset for PTB is between 40 and 50,^[2]^ with benign cases accounting for 35% to 64% and malignant cases accounting for 18% to 25%. According to an epidemiological study in Los Angeles, the prevalence of malignant PTB among women is approximately 0.21 per 1,00,000.^[3]^ Clinically, most patients present with painless masses in the unilateral breast that gradually increase over a long period, but a few may manifest as rapid enlargement of breast masses in a short period, even occupying the entire breast.^[4]^ Literature reports show that the average maximum diameter of PTB at presentation is 4 to 8 cm, and in rare cases, it can exceed 40 cm. Overall, PTB has a good prognosis, with a 5-year survival rate of over 90% after treatment.^[5,6]^ However, this tumor has a high risk of local recurrence, and the recurrence rate is closely related to the pathological type. The 5-year and 10-year local recurrence rates for benign PTB are approximately 8% and 13%, respectively, while the 10-year local recurrence rate for borderline or malignant PTB can exceed 30%.^[7]^ Malignant PTB has a high risk of metastasis, ranging from 16% to 25%, mainly through blood dissemination and rarely through lymph node metastasis (only about 5%). The most common sites of metastasis are the lungs (91%) and bones (39%).^[8]^ Due to the rarity of this type of tumor, there are relatively few large-scale studies on its etiology, diagnosis, treatment, and prognosis, and there are still many controversies regarding its treatment options.^[9]^ Lack of understanding of PTB can lead to delayed diagnosis and treatment, potentially allowing the condition to develop into a very difficult state.

This case report aims to document the management and outcome of a patient with breast phyllodes tumor, a rare but distinct type of breast tumor. The patient in this report presented with a palpable mass in the breast, which was subsequently diagnosed as a phyllodes tumor through a combination of clinical examination, imaging studies, and histopathological analysis. Surgical excision was the primary treatment modality, to achieve complete removal of the tumor while preserving as much healthy tissue as possible.

This report details a preoperative evaluation, surgical procedure, postoperative care, and follow-up of the patient. We also discussed the challenges encountered during the management of this case, including the difficulty in diagnosing phyllodes tumors and the need for a multidisciplinary approach to ensure optimal patient outcomes. By sharing this case report, we hope to contribute to the understanding of phyllodes tumors and improve the management of similar cases in the future.

## 2. Case presentation

**Figure 1.**
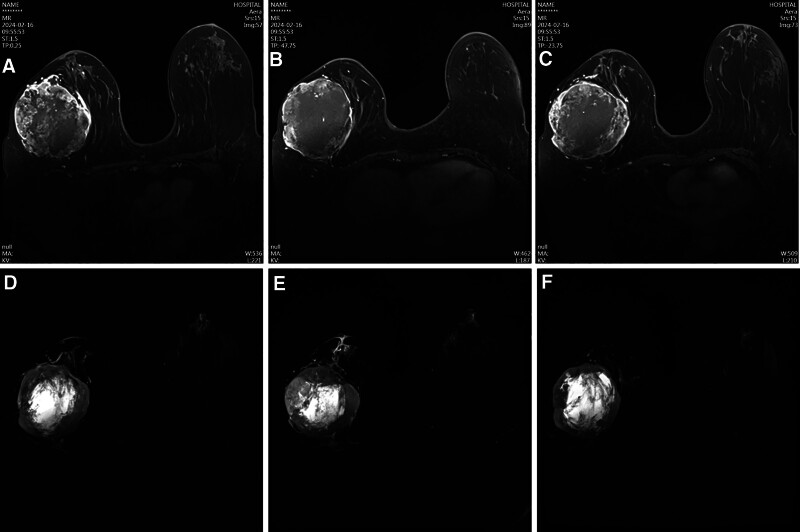
(A–F) MRI of the right breast tumor when the patient’s diagnosis. MRI = magnetic resonance imaging.

**Figure 2.**
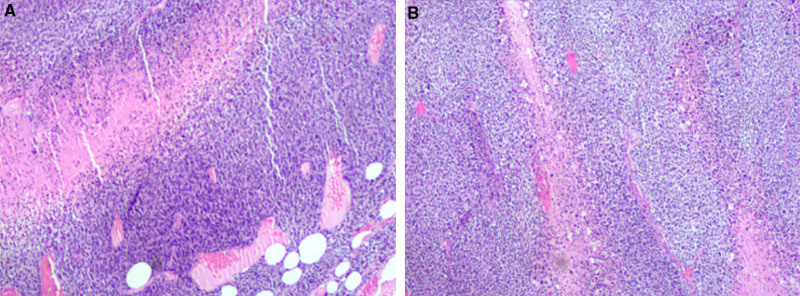
(A, B) The pathological results of the needle biopsy of the right breast tumor when the patient’s diagnosis.

The patient gave written informed consent for publication of this case report. The patient is a 50-year-old female who discovered a right breast mass 10 years ago, initially measuring only 2 to 3 cm. She promptly sought medical attention and treatment. Before hospital admission, she noticed a rapid enlargement of the right breast mass, without apparent provocation, to approximately 12 cm in size over a week. Upon outpatient consultation, the breast ultrasound revealed a large cystic-solid mass in the right breast, categorized as BI-RADS 4B. Breast magnetic resonance imaging with contrast (Fig. 1A–F) also showed a mass on the outer side of the right breast, classified as BI-RADS 4. Further, the patient underwent a biopsy of the right breast mass, with pathological findings indicating a neoplastic lesion with necrosis. Immunohistochemical analysis revealed positive expressions for Vimentin (+), CD34 (+), CK (+), Ki-67 (70%+), SMA (+), S-100 (+), Desmin (−), STAT6 (−), BCL2 (+), CD99 (+), CD31 (−), TLE (+), EMA (−), and CD10 (+; Fig. 2A, B). A contrast-enhanced computed tomography scan of the neck, chest, and abdomen excluded distant metastasis.

**Figure 3.**
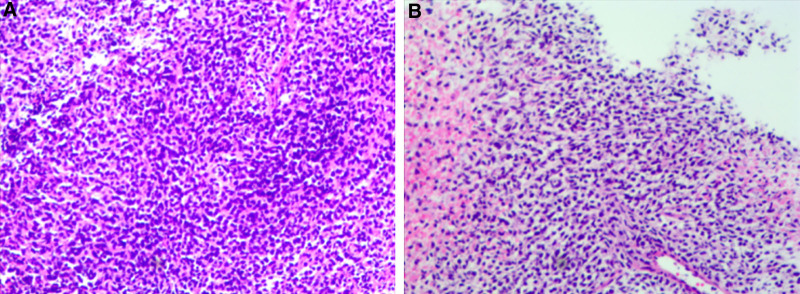
(A, B) The pathological results of the patient’s right breast tumor excision surgery after the operation.

Due to the large size of the mass, the patient underwent a right breast mastectomy. Postmastectomy pathological examination confirmed a malignant tumor of the right breast with necrosis. Morphological and immunohistochemical analysis suggested a malignant breast phyllodes tumor with excessive stromal growth, invasion of adipose tissue, and no tumor involvement at the nipple, skin margins, or localized proximity to the base margin (<5 mm). Eight lymph nodes were detected in the surrounding adipose tissue, with no evidence of tumor metastasis. Immunohistochemical analysis showed positive expressions for Vimentin (+), CK (+), Ki-67 (90%+), BCL2 (+), and CD99 (+), and negative expressions for CD34 (−), ER (−), PR (−), P53 (mutated), S-100 (−), and CK5/6 (−; Fig. 3A, B). The initial diagnosis was phyllodes tumor of the right breast, post-excision, staged as T4N0M0GX. The patient’s surgical incision has healed, and she is currently undergoing local radiotherapy at our center. The radiotherapy target volumes include gross tumor volume tumor bed (GTVtb; the original tumor bed area), clinical tumor volume (GTVtb expanded by 4 cm, with anatomical adjustments), and planning tumor volume (clinical tumor volume expanded by 0.5 cm). The prescribed doses are GTVtb 60Gy/25F and planning tumor volume 50Gy/25F. The key diagnostic and therapeutic milestones are summarized in Table 1. Serial imaging (every 3 months) demonstrated stable postradiation changes without suspicious lesions. Patient-reported outcomes indicated resolution of preoperative pain and maintained shoulder mobility. Ethical approval for this study was obtained from the Ethics Committee of Hebei University Affiliated Hospital.

**Table 1:** Clinical timeline of diagnosis and treatment.

Time point	Clinical event	Key findings/interventions
10 yr ago	The patient noticed a right breast mass	Initial size: 2–3 cm; no further action documented
1-wk preadmission	Rapid enlargement of the mass	Size increased to ~12 cm; prompted medical consultation
Day 1	Outpatient visit: breast ultrasound	BI-RADS 4B cystic-solid mass (Fig. 1A, B)
Day 3	Breast MRI with contrast	BI-RADS 4 mass on the outer right breast (Fig. 2A–F)
Day 5	Core needle biopsy of the mass	Pathology: neoplastic lesion with necrosis. IHC: Vimentin+, CD34+, Ki-67 (70%+), etc (Fig. 3A, B)
Day 7	CT scan (neck/chest/abdomen)	No distant metastasis confirmed
Day 10	Right mastectomy performed	Final pathology: malignant PTB with stromal overgrowth, negative margins, no nodal involvement
Post-op (4 wk)	Initiation of adjuvant radiotherapy	Target volumes: GTVtb (60 Gy), PTV (50 Gy) in 25 fractions
Follow-up	Regular monitoring	Incision healed; no recurrence reported at last follow-up

CT = computed tomography, GTVtb = gross tumor volume tumor bed, MRI = magnetic resonance imaging, PTB = phyllodes tumor of the breast, PTV = planning tumor volume.

## 3. Discussion

PTB is characterized by multifocal, often unilateral onset, prolonged duration, and rare bilateral involvement. It is often manifested as a painless mass within the breast.^[10]^ PTB progresses slowly in 25% to 40% of cases, but rapid growth may occur, particularly in borderline or malignant subtypes.^[10,11]^ The mass may exhibit skin ulceration, though it typically demonstrates expansive growth. PTB exhibits similar ultrasound and mammographic features to fibroadenoma, with well-defined solid nodules being the predominant finding. However, there is still some controversy regarding the surgical approach for borderline and malignant PTB.^[9,12]^ On T2-weighted imaging, PTB has a higher probability of internal cystic degeneration and increased signal intensity in the surrounding breast tissue compared to FA. The absence of enhanced intervals within the mass and the appearance of slit-like changes in the enhanced image are significantly correlated with higher histological grading of PTB. However, there is no significant statistical difference in the size, shape, margin, T1-weighted imaging and T2-weighted imaging signal enhancement (homogeneous or heterogeneous), or cystic changes among different types of PTB.^[13,14]^

Surgical treatment is the preferred treatment option for primary PTB. The surgical approach for benign PTB is similar to that for FA, with breast tumor resection being the main treatment option.^[15]^ The recurrence rate after surgery is not significantly correlated with the choice of surgical approach (tumor resection, local extended resection, or total mastectomy), but complete tumor resection and clean resection are essential.^[16–18]^ There is still much controversy regarding the surgical approach for borderline and malignant PTB, and the determination of the surgical margin. In terms of surgical approach, early researchers believe that total mastectomy can significantly reduce the risk of local recurrence compared to local tumor resection. Surgical treatment is the preferred treatment option for primary PTB.

PTB patients have a very low risk of axillary lymph node involvement, and there are reports that the rate of axillary involvement in malignant PTB is only 1% to 2%. Even for patients with clinically suspected axillary lymph node enlargement, the possibility of metastasis after surgery is still very low. Therefore, it is not recommended to routinely perform axillary lymph node staging surgery for PTB patients. If preoperative imaging studies (such as axillary lymph node ultrasound or PET-CT) suggest suspected metastasis in the axillary lymph nodes, the axillary lymph node biopsy should be performed to confirm the diagnosis. For patients with suspected metastasis, axillary lymph node dissection should be performed. For patients who cannot undergo axillary lymph node biopsy, sentinel lymph node or enlarged lymph node resection biopsy can be considered during surgery.^[9]^

For benign PTB patients, only surgical treatment and regular follow-up are required, and no other adjuvant therapy is needed. There has been controversy over whether adjuvant radiotherapy is necessary for patients with borderline or malignant PTB after surgery.^[19]^ Many recent studies have found that radiotherapy can improve the local control rate. The local recurrence rate of patients with positive margins after surgery is significantly higher than that of patients with negative margins,^[20]^ but the local recurrence rate and survival rate of patients with positive or negative margins and a width of less than 1 cm after radiotherapy are not statistically different from those of patients who only received surgery and have negative margins, indicating that radiotherapy can reduce the local recurrence rate of patients with difficult-to-obtain negative margins to some extent.^[21]^ Therefore, for patients who cannot achieve negative margins through local extensive resection or total mastectomy, postoperative radiotherapy can be considered in combination with the patient’s condition.^[22–24]^ In real-world studies, the proportion of patients undergoing postoperative radiotherapy for borderline or malignant PTB has increased year by year worldwide. The adjuvant radiotherapy rate for patients with total mastectomy after PTB has increased from 3% in 1980 to 28% in 2010.^[25]^ However, there is currently no report supporting that adjuvant radiotherapy can improve the overall survival of patients. The NCCN guidelines do not recommend adjuvant radiotherapy for new-onset PTB patients, regardless of their malignancy status.^[15]^ However, according to the European Society for Medical Oncology clinical practice guidelines for soft tissue sarcoma diagnosis and treatment, adjuvant radiotherapy can be considered for patients with low-grade or high-grade soft tissue sarcoma located on the body surface and with a maximum tumor diameter of >5 cm.^[26]^

There are few studies on whether adjuvant chemotherapy is necessary for malignant PTB patients after surgery. Morales-Vásquez et al conducted an observational study on adjuvant chemotherapy for malignant PTB patients after surgery, and the results showed that it did not improve the disease-free survival rate or overall survival rate.^[27]^ The results of a multicenter retrospective study reported by Neron et al in 2020 also showed that adjuvant chemotherapy did not significantly benefit patients with malignant PTB.^[28]^ There are currently no reports on targeted therapy for malignant PTB.^[29–31]^

There are currently no reports on the use of endocrine therapy for malignant PTB. Since ER and PR are only expressed in epithelial cells, the stromal cells that are considered malignant components do not express ER and PR. Therefore, endocrine therapy has certain limitations in the clinical treatment of PTB, and systemic treatment is only considered for patients with recurrent metastasis and advanced malignant PTB.

This study has several limitations. First, as a single-case report, the findings cannot be generalized to broader populations. Second, the 12-month follow-up period may be insufficient to evaluate long-term recurrence risks. Third, the radiotherapy regimen was empirically designed due to the absence of consensus guidelines, which may affect reproducibility. Future multicenter studies with larger cohorts are needed to validate these observations.

## Acknowledgments

The research team is deeply thankful to all study participants.

## Author contributions

**Methodology:** Yuge Ran.

**Validation:** Shuai Qie.

**Writing – review & editing:** Yuge Ran, Shuai Qie.

**Writing – original draft:** Fei Li, Shuai Qie.

## References

[1] Mishra SP Tiwary SK Mishra M Khanna AK. Phyllodes tumor of breast: a review article. ISRN Surg. 2013;2013:361469. 23577269 10.1155/2013/361469 PMC3615633

[2] Belkacémi Y Bousquet G Marsiglia H. Phyllodes tumor of the breast. Int J Radiat Oncol Biol Phys. 2008;70:492–500. 17931796 10.1016/j.ijrobp.2007.06.059

[3] Li GZ Raut CP Hunt KK Feng M Chugh R. Breast sarcomas, phyllodes tumors, and desmoid tumors: epidemiology, diagnosis, staging, and histology-specific management considerations. Am Soc Clin Oncol Educ Book. 2021;41:390–404. 34010054 10.1200/EDBK_321341

[4] Strode M Khoury T Mangieri C Takabe K. Update on the diagnosis and management of malignant phyllodes tumors of the breast. Breast. 2017;33:91–6. 28327352 10.1016/j.breast.2017.03.001

[5] Tan BY Acs G Apple SK. Phyllodes tumours of the breast: a consensus review. Histopathology. 2016;68:5–21. 26768026 10.1111/his.12876 PMC5027876

[6] Tremblay-LeMay R Hogue J-C Provencher L. How wide should margins be for phyllodes tumors of the breast? Breast J. 2017;23:315–22. 27901301 10.1111/tbj.12727

[7] Zurrida S Bartoli C Galimberti V. Which therapy for unexpected phyllode tumour of the breast. Eur J Cancer. 1992;28:654–7. 1317204 10.1016/s0959-8049(05)80119-4

[8] Barth RJ Jr. Histologic features predict local recurrence after breast conserving therapy of phyllodes tumors. Breast Cancer Res Treat. 1999;57:291–5. 10617306 10.1023/a:1006260225618

[9] Bogach J Shakeel S Wright FC Hong NJL. Phyllodes tumors: a scoping review of the literature. Ann Surg Oncol. 2022;29:446–59. 34296360 10.1245/s10434-021-10468-2

[10] Chen JJ Zhu I Patel A. Management of concurrent malignant phyllodes tumor and invasive breast carcinoma. Adv Radiat Oncol. 2024;9:101448. 38550370 10.1016/j.adro.2024.101448 PMC10965428

[11] Bartels SAL van Olmen JP Scholten AN. Real-world data on malignant and borderline phyllodes tumors of the breast: a population-based study of all 921 cases in the Netherlands (1989 -2020). Eur J Cancer. 2024;201:113924. 38364628 10.1016/j.ejca.2024.113924

[12] Li LJ Zeng H Ou B. Ultrasonic elastography features of phyllodes tumors of the breast: a clinical research. PLoS One. 2014;9:e85257. 24454830 10.1371/journal.pone.0085257 PMC3893177

[13] Duman L Gezer NS Balci P. Differentiation between phyllodes tumors and fibroadenomas based on mammographic sonographic and MRI Features. Breast Care (Basel). 2016;11:123–7. 27239174 10.1159/000444377 PMC4881274

[14] Abdul Hamid S Rahmat K Ramli MT. Radiopathological characteristics and outcomes of phyllodes tumor of the breast in Malaysian women. Medicine (Baltim). 2018;97:e11412. 10.1097/MD.0000000000011412 PMC6081195 30075507

[15] Gradishar WJ Moran MS Abraham J. NCCN Guidelines® Insights: Breast Cancer, Version 4.2023. J Natl Compr Canc Netw. 2023;21:594–608. 37308117 10.6004/jnccn.2023.0031

[16] Barth RJ Wells WA Mitchell SE Cole BF. A prospective, multi-institutional study of adjuvant radiotherapy after resection of malignant phyllodes tumors. Ann Surg Oncol. 2009;16:2288–94. 19424757 10.1245/s10434-009-0489-2 PMC5053421

[17] Choi N Kim K Shin KH. Malignant and borderline phyllodes tumors of the breast: a multicenter study of 362 patients (KROG 16-08). Breast Cancer Res Treat. 2018;171:335–44. 29808288 10.1007/s10549-018-4838-3

[18] Pezner RD Schultheiss TE Paz IB. Malignant phyllodes tumor of the breast: local control rates with surgery alone. Int J Radiat Oncol Biol Phys. 2008;71:710–3. 18234448 10.1016/j.ijrobp.2007.10.051

[19] Boutas I Kontogeorgi A Dimas D. Local recurrence for phyllodes tumours of the breast: systematic review and meta-analysis. Oncol Lett. 2022;24:353. 36168312 10.3892/ol.2022.13473 PMC9478605

[20] Chen C Huang X Xu Y Sun Q. Rethinking on the management strategy of malignant phyllodes tumor of the breast: an analysis based on the SEER database. Medicine (Baltim). 2023;102:e33326. 10.1097/MD.0000000000033326 PMC10036047 36961178

[21] Zhang G Yang P Zeng J Wei C. Effect of radiation therapy on patients with stage T3 or T4 malignant phyllodes tumors: a retrospective observational study based on SEER. J Cancer Res Clin Oncol. 2023;150:2. 38153521 10.1007/s00432-023-05517-0 PMC10754728

[22] Boutrus RR Khair S Abdelazim Y. Phyllodes tumors of the breast: adjuvant radiation therapy revisited. Breast. 2021;58:1–5. 33865208 10.1016/j.breast.2021.03.013 PMC8079270

[23] Mitus JW Blecharz P Jakubowicz J Reinfuss M Walasek T Wysocki W. Phyllodes tumors of the breast. The treatment results for 340 patients from a single cancer centre. Breast. 2019;43:85–90. 30521986 10.1016/j.breast.2018.11.009

[24] Zhang H Tang S Biskup E. Long-term survival after diverse therapeutic modalities in malignant phyllodes tumors of the breast. Technol Cancer Res Treat. 2022;21:15330338221121086. 36000314 10.1177/15330338221121086 PMC9425899

[25] Adesoye T Neuman HB Wilke LG Schumacher JR Steiman J Greenberg CC. Current trends in the management of phyllodes tumors of the breast. Ann Surg Oncol. 2016;23:3199–205. 27334214 10.1245/s10434-016-5314-0 PMC5021443

[26] Gronchi A Miah AB Dei Tos AP. Soft tissue and visceral sarcomas: ESMO-EURACAN-GENTURIS clinical practice guidelines for diagnosis, treatment and follow-up(☆). Ann Oncol. 2021;32:1348–65. 34303806 10.1016/j.annonc.2021.07.006

[27] Morales-Vásquez F Gonzalez-Angulo AM Broglio K. Adjuvant chemotherapy with doxorubicin and dacarbazine has no effect in recurrence-free survival of malignant phyllodes tumors of the breast. Breast J. 2007;13:551–6. 17983394 10.1111/j.1524-4741.2007.00510.x

[28] Neron M Sajous C Thezenas S; French Sarcoma Group (GSF-GETO). Surgical margins and adjuvant therapies in malignant phyllodes tumors of the breast: a multicenter retrospective study. Ann Surg Oncol. 2020;27:1818–27. 31989361 10.1245/s10434-020-08217-y

[29] Valenza C De Pas TM Gaeta A. Primary malignant phyllodes tumors of the breast: a retrospective analysis from a referral center. Eur J Cancer. 2024;196:113423. 37977104 10.1016/j.ejca.2023.113423

[30] Samii E Hurni Y Huber D. Management and outcomes of metastatic and recurrent malignant phyllodes tumors of the breast: a systematic literature review. Eur J Breast Health. 2023;19:191–200. 37415652 10.4274/ejbh.galenos.2023.2023-3-2 PMC10320634

[31] Goodwin B Oyinlola AF Palhang M. Metastatic and malignant phyllodes tumors of the breast: an update for current management. Am Surg. 2023;89:6190–6. 37611540 10.1177/00031348231198114

